# Efficacy and acceptability of using wearable activity trackers in older adults living in retirement communities: a mixed method study

**DOI:** 10.1186/s12877-022-02931-w

**Published:** 2022-03-21

**Authors:** Zhanjia Zhang, Bruno Giordani, Alayna Margulis, Weiyun Chen

**Affiliations:** 1 Department of Physical Education, Peking University, Beijing, 100871 China; 2 School of Kinesiology, University of Michigan, 1402 Washington Heights, 3145 OBL, Ann Arbor, MI 48109 USA; 3 Department of Psychiatry, University of Michigan, Ann Arbor, MI 48109 USA

**Keywords:** Technology, Self-regulation, Exercise, Retirement community

## Abstract

**Background:**

Wearable activity trackers hold the potential for enhancing health and fitness, but the use of wearable activity trackers has remained largely unexplored in older adults. The purpose of the current study was to examine the effectiveness and acceptability of wearable activity trackers for promoting physical activity (PA) in older adults living in retirement communities.

**Methods:**

Forty older adult participants (mean age = 85.4 years) used a wearable activity tracker (Fitbit InspireHR) for 12 weeks. Participants were provided with personalized activity goals and weekly feedback of PA during the 12 weeks. The main outcomes were daily step counts collected at baseline and the end of the intervention, and participants’ experiences of using the wearable activity tracker assessed after the 12-week intervention through an 8-item questionnaire and individual interviews.

**Results:**

Participants used the activity tracker on 97.5% of measured days and had an average increase of 900 steps/day (*p* < 0.001). The Acceptance questionnaire revealed that the wearable activity tracker was acceptable, useful, and easy to use. Participants found that wearable activity trackers helped improve self-awareness and motivation of PA but reported a few concerns regarding the comfort of wearing the activity trackers and the ease of reading visual feedback.

**Conclusions:**

Wearable activity trackers lead to a small but significant increase of PA and are perceived as acceptable and useful in older adults. Given the rapidly growing older population, wearable activity trackers are promising tools that could be used in large-scale interventions to improve PA and health in older adults.

**Trial registration:**

Registered on Clinicaltrials.gov # NCT05233813 (Registered on 10/02/2022).

**Supplementary Information:**

The online version contains supplementary material available at 10.1186/s12877-022-02931-w.

## Background

Regular physical activity (PA) is associated with various physical and mental health benefits in older adults, including lower risk of mortality, better cardiovascular health, improved sleep quality, and reduced risk of functional disability [1]. The 2018 Physical Activity Guidelines for Americans recommends that older adults participate in at least 150 min of moderate-intensity PA or 75 min of vigorous-intensity PA per week to obtain substantial health benefits [2]. Despite the well-documented links between PA and health, only 27% of older adults aged 65 and older meet the PA guidelines according to the National Health and Nutrition Examination Survey [3], and this number drops to 2.5% when PA is objectively assessed by accelerometers [4]. Therefore, effective interventions that aim to increase PA levels in the older population are warranted.

Among many types of PA, walking is identified as the favorite type of exercise among older adults [5]. Interventions targeting walking are most likely to be effective in changing PA behaviors in older adults [6]. As a cost-effective type of exercise, it is estimated that 10% of the adult population participating in a regular walking program could save $5.6 billion in heart disease costs [7]. Therefore, effective interventions aiming at increasing the walking among older adults may translate into reduced health care costs.

Interventions designed to increase PA usually incorporate various behavior change techniques [8]. A systematic review examined the effects of 26 behavior change techniques using a meta-regression and found that self-monitoring had the greatest individual effect in changing PA [9]. It was also found that interventions combining self-monitoring with at least one other technique of self-regulation (e.g., goal setting, providing feedback) were more effective in promoting PA than other interventions [9]. Self-monitoring is a key component of self-regulation based on the Social Cognitive Theory [10]. According to this theory, self-monitoring one’s performance helps individuals set realistic goals and assess their progress toward these goals [10]. Self-monitoring one’s PA also enhances one’s self-efficacy for PA, which is a direct predictor of participation in PA [11].

Wearable activity trackers have been increasingly used as a self-monitoring tool in PA interventions and incorporate multiple behavior change techniques. A recent study examined the behavior change techniques implemented in 13 commercially available activity trackers and found that self-monitoring and feedback were the mostly incorporated techniques among these activity trackers [12]. Wearable activity trackers monitor an array of PA indicators (e.g., step counts, distance walked, calories burned) and provide real-time feedback of these data for users. Unlike traditional hip-worn pedometers which require users to manually record daily step counts for long-term tracking, recent advances in technology allow wearable activity trackers to transmit and store data wirelessly through internet- or mobile- based applications [12]. Previous interventions using wearable activity trackers as a tool for promoting PA have shown promising results in various populations. For example, a randomized controlled trial conducted among overweight and obese adults showed that participants who wore a wearable activity tracker for 6 weeks increased daily step counts by 1266, while the attention control group showed no increase in daily step counts [13].

Interventions integrating the appropriate use of wearable activity trackers to promote PA in older adults has considerable public health importance for the aging society. First, as PA is associated with various health outcomes and the older adults are more universally sedentary than any other age group [14], wearable activity tracker-based interventions have the potential to prevent or slow-down the age-related declines in physical and cognitive functioning. Second, older adults are the fastest growing segment of the population regarding Internet use and smartphone ownership [15], which facilitates the potential adoption of wearable activity trackers in their daily lives. However, only a handful of intervention studies have used wearable activity trackers to promote PA in older adults. Cadmus-Bertram and colleagues [16] conducted a 16-week wearable activity tracker-based intervention and found it significantly increased the daily step counts by 789 among postmenopausal women. In contrast, another randomized controlled trial showed that a 24-week intervention combining wearable activity trackers with exercise counseling did not change the PA level in sedentary and overweight older adults [17]. Since the findings from previous studies are mixed and limited, more research is needed to investigate the effectiveness of wearable activity tracker-based interventions and understand how to best implement these interventions in older adults. Such information will contribute to informing the design and implementation of effective interventions to promote PA among the older population in future studies. As far as we know, only a few studies have examined the acceptability of wearable trackers in older adults and most of them were conducted within a relatively short period [18, 19].

The purpose of the current study was to examine the effectiveness and acceptability of wearable activity trackers combined with self-regulatory techniques for promoting PA in older adults living retirement communities. It was hypothesized that using wearable activity trackers combined with self-regulatory techniques (e.g., goal setting, self-monitoring, and feedback) would significantly improve the PA level and would be considered to be positive and acceptable among older adults. The majority of the older adults living in retirement communities were physically inactive as less than 15% of them meet the PA guidelines. The preliminary evidence of the current study would better inform how wearable activity trackers can help this population become more active.

## Methods

### Participants

Participants were 41 older adults voluntarily participating in a 12-week multicomponent PA intervention as part of a two-arm quasi-experimental trial. The multicomponent intervention had two major parts: wearing activity trackers combined with self-regulatory techniques and attending group exercise lessons (45-min each lesson, three lessons per week). The current study only focused on the former part.

Between May 2019 and August 2019, we recruited individuals from two retirement communities in southeast Michigan. Flyers were posted on community message boards and a 60-min on-site presentation was delivered to inform the procedures and benefits of participating in this study. Potential participants were screened for study eligibility using the criteria including: 1) aged 65 or older, 2) able to speak and read English fluently, 3) able to walk for 10 ft without human assistance, 4) scored 3 or greater in the Mini-Cog test, a screening test for cognitive impairment in older adults [20]. A total of 41 eligible individuals (mean (SD) age = 85.4 (5.1) years, 80.5% females) were voluntarily assigned to the intervention group in this study. All participants provided written informed consent before the study.

### Intervention

#### Activity tracker training and wearing protocols

Each participant received a Fitbit (model InspireHR) activity tracker at the beginning of the study. The Fitbit activity tracker is a commercially available and non-invasive activity tracker worn similar to a watch on the wrist. A built-in accelerometer captures body movement and then is converted to several PA indicators such as steps, distance, and floors climbed. The activity tracker provides real-time visual feedback of these PA indicators for users. To minimize the potential barriers of using the activity tracker, participants were given a one-hour training before the intervention on how to operate the activity tracker, navigate its basic functions, and charge the battery. In addition, ongoing support was also provided by the research team to allow participants to report any issues in using the activity tracker during the 12-week intervention. Participants were asked to wear the activity tracker from Monday to Friday during their wake hours every week throughout the intervention and encouraged to frequently check their PA indicators on the activity tracker.

#### Goal setting

Setting an activity goal is an important predictor of increased PA [21]. Each participant was guided to set an individualized step-count goal with a plan for increasing average daily steps by 500 to 1500 steps every 2 weeks based on their baseline step counts, which eventually led to achieving or maintaining 7500 steps a day by the end of the intervention. For example, if a participant’s average daily step counts in the previous 2 weeks ranged from 2500 to 3000, his or her goal for the next 2 weeks would be 4000 steps per day. If a participant did meet the previous goal, the subsequent goal of daily steps would not be increased. The ultimate goal of 7500 steps a day was set based on a recent study showing that the risk of mortality in older adults decreases as daily step counts increase before leveling at 7500 steps per day [22]. Every 2 weeks, each participant received a one-page weekly PA goal sheet to indicate their activity goals for the next 2 weeks.

#### Weekly individualized feedback

Each participant received weekly individualized feedback summarizing their daily step counts during the previous week. Although the activity tracker is associated with a mobile app that can track PA indicators, the majority of participants in the current study did not have access to a smartphone. Therefore, we created a Fitbit account and password for each participant. We collected each participant’s activity tracker every weekend and synced the PA data to each participant’s online account. The participants’ daily step counts during the previous week were exported using the Fitabase analytics system (Small Steps Labs, San Diego, CA, USA) which allowed us to manage the activity tracker data for multiple participants. A personalized printout of feedback was reported and the activity trackers were returned to each participant before every Monday morning.

### Measures

#### Demographics

Demographic information including age, gender, race, marital status (married/not married), and education (not graduate high school/high school graduate/bachelor degree or higher) was obtained at baseline using a questionnaire.

#### Daily step counts

Daily step counts were objectively measured by the activity tracker. The average daily step counts during the first week of the intervention were calculated as the baseline daily step counts. This was because the goal setting and feedback started in the second week of the intervention. The average daily step counts during the last 2 weeks of the intervention were computed as the endpoint daily step counts. Days in which the daily step counts less than 500 were considered non-wearing days and thus were excluded in the calculation [23]. The Fitbit activity tracker has been demonstrated to accurately track steps in older adults [24]. Data of daily step counts were stored and downloaded from the Fitabase analytics system.

#### Acceptance of activity trackers

At the end of the intervention, each participant completed an in-person assessment including a questionnaire and a semi-structured individual interview regarding their acceptance of using the Fitbit activity tracker. The 8-item Acceptance questionnaire with a 5-point rating scale was adapted from a previous study in which it was used in adults with chronic illness [25]. The questionnaire mainly focused on the perceived usefulness and perceived ease-of-use based on the Technology Acceptance Model [26]. Participants rated on a 5-point Likert scale to indicate the extent to which they endorse each item from 1 (strongly disagree) to 5 (strongly agree). The internal consistency was good in the current study (Cronbach’s alpha = 0.90). The detailed items were listed in Table 2.

A semi-structured individual interview was conducted with each participant to collect further information regarding their acceptance of using the activity tracker. The interview consisted of four questions asking participants to indicate what they liked or disliked about the activity tracker, what improvement could be made to make the activity tracker more useful to them, and whether they would continue using the activity tracker to monitor their activities. Each interview lasted 5 to 10 min and was audio-taped using a recorder. The interviews were conducted in a conference room in the retirement communities. Each interview was transcribed verbatim for later analysis.

### Data analysis

Baseline characteristics were described using descriptive statistics with mean and standard deviation for numerical variables and with frequency and percentage for categorical variables. For the Acceptance questionnaire assessing the acceptance of using the activity tracker, the average score of each item was calculated. A paired t-test was conducted to examine the change of average daily step counts from baseline to endpoint with a significance level of *p* < .05 (no violation of normality was detected for the baseline daily step counts and endpoint daily step counts by checking the skewness and kurtosis). Cohen’s d was calculated as an index of effect size by dividing the mean difference of daily step counts between baseline and endpoint by the standard deviation of baseline daily step counts. All quantitative analyses were performed in SPSS (version 26).

The interview transcripts were analyzed using the thematic analysis [27]. We chose thematic analysis because it does not require a pre-existing theoretical framework yet to organize and describe data in rich detail. First, the transcripts were read and re-read to generate initial codes. Second, the codes were collated and similar codes were gathered to form potential themes. Following this process, the themes were refined by reviewing the codes in each theme and connecting themes with each other to validate the similarities and dissimilarities. The qualitative data analysis was performed using the NVivo (version 12).

## Results

### Baseline characteristics

One participant dropped out of the study in the first week of intervention, reporting dissatisfaction on the accuracy of the activity tracker in recording step counts. The remaining 40 participants completed the endpoint assessment and were included in the final analysis. The baseline characteristics of these participants were presented in Table 1. The average age of participants was 85.4 years (SD = 5.2) and more than half (55%) of them were 85 years or older. The majority of participants were female (80%), had graduated from high school or above (95%), and were not married (67.5%). All of the participants were Whites.

**Table 1:** Demographic characteristics of participants

	*n* (%)
Age	
75–84	18 (45%)
> =85	22 (55%)
Gender	
Male	8 (20%)
Female	32 (80%)
Education	
Did not graduate high school	2 (5%)
High school graduate	21 (52.5%)
Bachelor degree or higher	17 (42.5%)
Marital Status	
Married	13 (32.5%)
Not married	27 (67.5%)
Race	
White	40(100%)

### Effectiveness

**Fig. 1:**
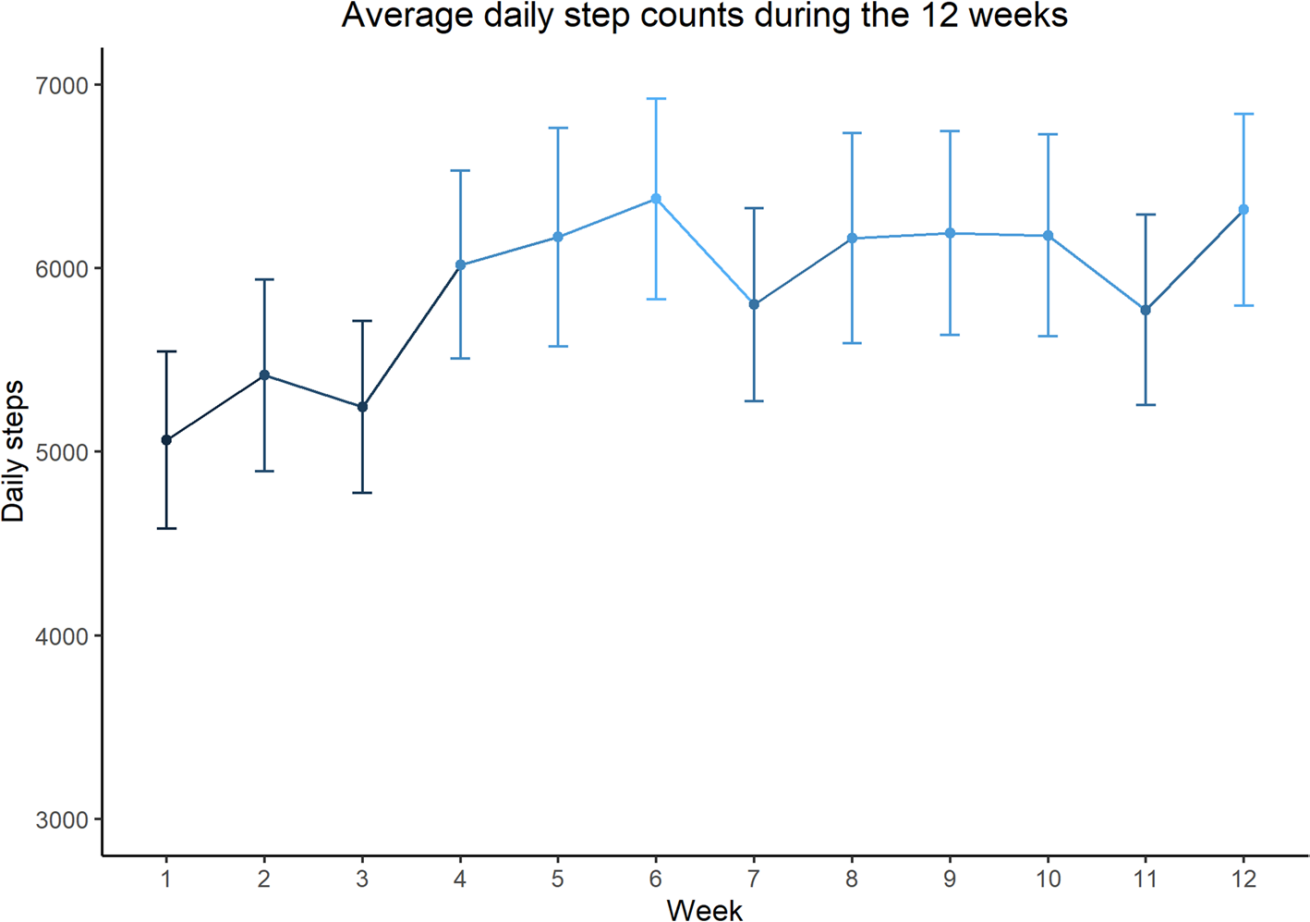
Average daily step counts over the 12 weeks. Note: error bars denote standard error of the mean

The average daily step counts of participants were 5063 ± 3049 at baseline and 5963 ± 3244 at the end of the intervention. Figure 1 shows the changes of average daily step counts over the 12 weeks such that average daily step counts peaked at the sixth week and maintained relatively stable during the rest of the weeks. The paired t-test revealed that this average increase of 900 steps per day was significant (*t* = 3.56, *p* < 0.001). Cohen’s d was equal to 0.3, indicating a small effect size [28].

### Acceptability

#### Adherence to wearing the activity tracker

Data indicated that participants wore the activity trackers during 97.5% of the required days, suggesting high compliance with the intervention. One participant was replaced with a new activity tracker in the third week of the intervention due to a battery issue. Another two participants reported that the activity trackers did not display the time correctly in the fourth week and fifth week. Another participant reported having trouble navigating to the main screen of the activity tracker. Our research team fixed these issues immediately after receiving the request for assistance. No other technical issues were reported during the intervention.

#### Acceptance questionnaire

The average score of each item of the Acceptance questionnaire ranged from 3.83 for the item “I found the Fitbit clear and understandable to use” to 4.26 for the item “The weekly Fitbit’s feedback (provided by the research team) was helpful” (Table 2). The average score was 4.00 for item 1 measuring the overall satisfaction with the wearable activity tracker. The mean score of all items was 4.00. The results of the Acceptance questionnaire indicated that participants considered the wearable activity trackers to be helpful and acceptable.

**Table 2:** Items and scores of the Acceptance questionnaire

Item	Mean (score range 1–5)	SD
Overall, I was satisfied with the Fitbit.	4.00	0.83
Using the Fitbit kept me more active.	4.10	0.71
Using the Fitbit helped me set activity goals.	3.92	0.83
Setting the activity goals helped me be more active.	4.03	0.64
Using the Fitbit helped me reach my activity goals.	3.90	0.79
The weekly Fitbit’s feedback (provided by the research team) was helpful.	4.26	0.68
I found the Fitbit clear and understandable to use.	3.83	0.75
Overall, the Fitbit is easy to use.	3.95	0.75

#### Individual interviews

Three overarching themes regarding the attitudes towards the wearable activity tracker emerged from the analysis of the interview transcripts. These were liked features, disliked features, and additional suggestions. Sample quotes for each theme can be found in the supplementary materials.

##### Theme 1: liked features

Self-awareness was the most frequently mentioned theme in the interviews. When asked what they liked about the activity tracker, 20 participants (50%) indicated that the activity tracker made them more aware of their walking/daily steps. Nine participants (22.5%) also mentioned that the activity tracker helped them monitor their heart rate.

Checking the visual feedback from the activity tracker facilitated their PA behavior change through goal achievement. Eleven participants (27.5%) reported that being aware of their PA helped them achieve their activity goals more effectively. Note that this was different from awareness in that it evidently involved activity goals, although the goal achievement could be a consequence of self-awareness.

##### Theme 2: disliked features

When asked about the disliked features of the activity tracker, eight participants (20%) reported that wearing the activity tracker was sometimes uncomfortable. The uncomfortable experience was mainly because the watchband of activity tracker was too tight or the skin touch with the metal part of the activity tracker was annoying. Seven participants (17.5%) reported that they found it hard to read the visual feedback mainly because the numbers displayed on the activity tracker were too small.

##### Theme 3: additional suggestions

When asked about the possible improvement that could make the activity tracker more useful to them, 12 participants (30%) expressed their interest in exploring other functions of the activity tracker, such as tracking water intake and sleep. The activity tracker showed the potential for long-term use based on the interviews. The majority of participants (95%) expected that they would continue using the activity tracker. Only two participants (5%) reported they would not continue using the activity tracker. One indicated that the activity tracker was perceived inaccurate and the other indicated that he was not interested in the feedback the activity tracker provided.

## Discussion

The current study examined the efficacy and acceptability of wearable activity trackers in promoting PA in older adults living in retirement communities. Findings from objectively measured PA indicated that the wearable activity tracker was effective in promoting PA in older adults. Results from the acceptance questionnaires and individual interviews indicated that the wearable activity tracker was an acceptable tool for older adults to self-track their PA.

Of the 40 participants who wore the activity tracker for 12 weeks and received weekly feedback and personalized goals, the average daily step counts increased by 900, equivalent to 18% of baseline daily step counts. Although the effect size (Cohen’s d = 0.3) was small according to the commonly used interpretation, such an increase may be associated with significant health benefits for this population [29]. The magnitude of the intervention effect was similar to a few previous studies. In their study involving older women aged 60 to 78 years, Koizumi and colleagues [30] found that a 12-week intervention combining waist-worn activity trackers with bi-weekly feedback and progressive goal setting significantly increased participants’ daily step counts by 16%. In another study, Cadmus-Bertram and colleagues [16] conducted a 16-week Fitbit-based intervention with a fixed activity goal of 10,000 steps per day in postmenopausal women. Participants in their study increased daily step counts by 13.3%. The findings from the current study support these studies showing that self-monitoring of PA combined with personalized goal setting and feedback led to a small but significant increase of PA in older adults.

Participants in the current study generally found the activity tracker to be satisfactory, useful, and easy to use after using it for 12 weeks. The activity tracker data were available for the majority of measured days, indicating the actual use of activity tracker was high. Results of the Acceptance questionnaire indicate that participants endorse more on the usefulness than the ease-of-use of the activity tracker, which are two key components of the Technology Acceptance Model. This was also reflected by a few reported cases of technical issues during the 12-week use of the activity tracker, as well as barriers identified in the interviews. The ease-of-use might be a priority focus for future interventions aiming at facilitating the adoption of wearable activity trackers in the older population. Although the current study included one session for guiding participants to use the activity tracker before the intervention and provided ongoing technical support, this might not be sufficient to eliminate technical barriers for older adult users. Future research may consider including more training sessions throughout the intervention.

The Acceptance questionnaire also shows that participants rated most highly on the weekly feedback of step counts provided by our research team. This suggests that feedback over a period of time provides additional value in increasing older adults’ perceived usefulness of the activity tracker on top of the instant feedback. It should be noted that feedback over days or weeks could not be displayed on the activity tracker. Users need to sync their activity tracker data with a smartphone or computer via Bluetooth and access a mobile app or Website to review their past activity tracker data, which may cause additional effort for older adults. Nevertheless, the rapidly increasing number of smartphone owners in the older population makes the large-scale adoption of the activity tracker in older adults promising in the future.

Self-awareness is the most prominent theme identified in the interviews. Being able to check their step counts throughout the day prompts changes in their PA behavior. This is in line with a few previous studies evaluating the use of activity trackers in older adults. In the study by Mercer and colleagues [25] involving older adults with chronic illness, participants used four wearable activity trackers with each for 3 days and perceived that activity trackers helped them become more aware of their activity levels. That the activity tracker increases self-awareness and motivation of PA was also identified in men with prostate cancer who wore the Fitbit activity tracker for 3 weeks [19] and breast cancer survivors who used two to three different activity trackers with each for 2 weeks [18]. The current study supports these findings by expanding the length of using activity trackers to 12 weeks. Besides the increased self-awareness of PA, participants in the current study also liked having the ability to check their heart rate throughout the day. This was an unexpected finding because the heart rate was not the main focus of the current study. However, it has significant implications for future interventions such that integrating health indicators into the wearable activity tracker may facilitate its use in the older population. Manufacturers may consider improving the usefulness of the wearable activity tracker by expanding its capability of self-monitoring to other indicators that are critical to older adults, such as blood glucose and blood pressure, if it is technically feasible.

Comfort and ease of reading are important to older adult users of activity trackers, as several participants in the current study reported negative experiences in these two aspects. We also from time to time observed that some participants took up to a few minutes to wear the activity tracker and adjust the watchband. When developing future activity trackers for older adults, it should be kept in mind that older adults generally have functional declines such as impaired vision and weakened muscle strength [31]. Wearable activity trackers designed for older adults must be comfortable to wear, easy to operate, clear to understand, and avoid arousing unpleasant feelings.

The interviews also revealed participants’ interest in using other functions of the activity tracker. Future research should investigate how to integrate all functions and features of the activity tracker to optimize PA interventions. For example, although not used in the current study, the mobile app and Website of the Fitbit activity trackers allow users to have a virtual social community and share their PA data with others in the social community. Competing with important others such as family and friends may also increase their motivation for PA, as one participant (P9) in the present study indicated that “*it is kinda like a game in a way because it encourages my husband and me to do more activity.*”

Although the current study demonstrates that the activity tracker-based intervention is promising in promoting PA in older adults, several limitations should be noted. First, participants in the current study were relatively well-educated and consisted of all White, which limits our generalizability of the study results. Including older adults with different race/ethnicities and educational backgrounds in future studies would contribute to more diverse opinions toward the use of activity trackers in older adults. Second, our findings regarding the significant intervention effect in increasing daily steps might be conservative for two reasons. On the one hand, the activity tracker in the current study was both an intervention component and an assessment tool of baseline daily step counts. Therefore, participants might be inadvertently exposed to a certain extent of the intervention during the baseline measure of daily step counts. This might in turn lead to the underestimation of the intervention effect, although evidence shows owning the activity tracker alone is unlikely to elicit behavior change [32]. On the other hand, the current study did not exclude participants who were already physically active. We suggest future studies target specifically inactive older adults and objectively measure the PA (e.g., using accelerometers) before the activity tracker is given to participants. Third, it is possible that participants may already hold a positive attitude towards the activity tracker before the intervention. Future studies should seek to examine the effects of activity trackers on PA among older adults who have low interest in using wearable activity trackers. Fourth, we did not conduct follow-up evaluations regarding the long-term use of the activity tracker, especially after stopping providing the weekly feedback. Nevertheless, we believe it is promising for continuous long-term use of the activity tracker by the participants because all but two of them expressed intention of continuing using the activity tracker to monitor their activities. Last, the current study failed to include a control group. Additional well-designed randomized controlled trials can help rule out confounders that may influence the efficacy and acceptability of using wearable activity trackers.

Despite these limitations, the findings of the current study have important implications for policy and health promotion in the older population. By conducting both quantitative and qualitative approaches, we demonstrated the feasibility and acceptability of using wearable activity trackers in older adults. Our study supported that older adults (even those 85 years or older) can utilize wearable activity trackers to effectively improve their PA levels. Given that the technology use among older adults is increasing rapidly [15], it is promising to integrate wearable activity trackers in large-scale health promotion programs. Wearable activity trackers can be incorporated into healthcare systems and clinical practice to provide healthcare professionals with accurate and real-time information about older adults’ activity levels. The barriers of using wearable activity trackers could be overcome by increasing educational sessions and making the presentation and interpretation of the activity data easier for older adults. It should be noted that our study was conducted before the COIVD-19 pandemic. The findings of the current may have greater significance for the healthcare delivery system in a time of social distancing as older adults are more vulnerable to COVID-19 than other age groups [33].

## Conclusion

The current study provides preliminary evidence that activity trackers, combined with behavior change techniques, are effective in promoting PA and acceptable to the older population. Older adults manifest high adherence to wearing and using the activity tracker, find the activity tracker to be helpful in enhancing self-awareness and PA motivation, and express intention for continuing using the activity to monitor their activities. Future designs of activity trackers targeting older adults could make the interface easier to read and require less fine motor skills to operate the activity tracker. In light of the rapidly growing older population, wearable activity trackers have significant potential to be a cost-effective and sustainable tool for promoting PA and improving health for older adults.

## Supplementary Information

**Additional file 1: Supplementary File.** Themes and Sample Quotes from Individual Interviews.

## Data Availability

The datasets generated and/or analyzed during the current study are not publicly available but are available from the corresponding author on reasonable request.

## Abbreviation

PA: Physical activity
