# Supplementary material for: Efficacy and acceptability of using wearable activity trackers in older adults living in retirement communities: a mixed method study
Source: BMC Geriatr. 2022 Mar 21;22:231. doi: 10.1186/s12877-022-02931-w (PMC8935620; doi:10.1186/s12877-022-02931-w)
Supplement: Supplementary file 1 — Additional file 1: Supplementary File. Themes and Sample Quotes from Individual Interviews. [file 12877_2022_2931_MOESM1_ESM.docx]

**Supplementary File**

Themes and Sample Quotes from Individual Interviews

| Themes | Subthemes | Quotes |
| --- | --- | --- |
| Liked features | Self-awareness of daily steps | Well I could find out how many steps I had walked because I don’t purposefully go out and walk on the track or that sort of thing. So to know I’m getting some sort of walking in is good. (P33) |
|  | Self-awareness of heart rate | Even that I'm not sure how good that would be. It works mainly on your heartbeat more than anything else. Going through your pulse doesn’t mean that you are walking or running or swimming. Your pulse could be fast just because you are watching a program on TV that is getting you excited or you see a handsome man walking down the road and get excited. (P13) |
|  | Goal achievement | I could get my heartbeat, the date of course and the number of steps. The calories I didn’t pay much attention to but the number of steps was important. I tried to gain my 7,500 and I didn’t do it all the time. Like Monday I was sick and didn’t get much out of bed. (P14) |
| Disliked features | Uncomfortable experiences | It sticks. It doesn’t move and that’s part of the problem. My cuffs sometimes don’t go over it. I have to keep it loose so it doesn’t squeeze me. (P39) |
|  | Difficulty in reading visual feedback | If it were bigger so I could see it better. And I think if I had a bigger one it would be on more of my arm and record more accurately. (P25) |
| Additional suggestions | Exploring other functions | I don’t know. I’d like some of the other settings. Like how much water you drink and so forth you could probably program it for that. I guess it has how many calories I’ve burned. (P20) |
|  | Potential for long-term use | Oh yes, I definitely will. I have blood clots in my lungs and the doctor said if I don’t exercise I’ll have more. (P24) |
